# Sedimentation Patterns of Toxin-Producing *Microcystis* Morphospecies in Freshwater Reservoirs

**DOI:** 10.3390/toxins5050939

**Published:** 2013-05-03

**Authors:** Samuel Cirés, Lars Wörmer, David Carrasco, Antonio Quesada

**Affiliations:** Departamento de Biología, Universidad Autónoma de Madrid, C/Darwin, 2, Madrid 28049, Spain; E-Mails: samuel.cires@uam.es (S.C.); lwoermer@marum.de (L.W.); david.carrasco@inia.es (D.C.)

**Keywords:** *Microcystis*, microcystins, hepatotoxins, sedimentation, settling, water reservoirs, annual cycle

## Abstract

Understanding the annual cycle of *Microcystis* is essential for managing the blooms of this toxic cyanobacterium. The current work investigated the sedimentation of microcystin-producing *Microcystis* spp. in three reservoirs from Central Spain during the summer and autumn of 2006 and 2007. We confirmed remarkable settling fluxes during and after blooms ranging 10^6^–10^9^ cells m^−2^ d^−1^, which might represent 0.1%–7.6% of the organic matter settled. A comprehensive analysis of the Valmayor reservoir showed average *Microcystis* settling rates (0.04 d^−1^) and velocities (0.7 m d^−1^) that resembled toxin settling in the same reservoir and were above most reported elsewhere. *M. aeruginosa* settling rate was significantly higher than that of *M. novacekii* and *M. flos-aquae*. Despite the fact that colony sizes did not differ significantly in their average settling rates, we observed extremely high and low rates in large colonies (>5000 cells) and a greater influence of a drop in temperature on small colonies (<1000 cells). We found a 4–14 fold decrease in microcystin cell quota in settling *Microcystis* of the Cogotas and Valmayor reservoirs compared with pelagic populations, and the hypothetical causes of this are discussed. Our study provides novel data on *Microcystis* settling patterns in Mediterranean Europe and highlights the need for including morphological, chemotypical and physiological criteria to address the sedimentation of complex *Microcystis* populations.

## 1. Introduction

The colonial genus *Microcystis* (Chroococcales) is one of the most common bloom-forming cyanobacteria in freshwater bodies and a major producer of the hepatotoxins microcystins (MCs) worldwide [1,2], thus posing a great concern for water quality management.

Understanding the annual life-cycle of *Microcystis* is essential for developing water management strategies to delay or minimize the blooms of this harmful cyanobacterium [3]. *M. aeruginosa*, the best known *Microcystis* spp., has a meroplanktonic behavior with an annual life-cycle consisting of 4 stages for the temperate regions [4,5,6]: pelagic growth that occurs mostly in summer; sedimentation of the pelagic population to the bottom sediments in autumn; overwintering as benthic or small pelagic populations; and reinvasion of the water column (recruitment) in spring, returning to the beginning of the cycle. This cycle has been subject of a number of field studies beginning from the early 1980s to the present [4,5,7,8,9,10], including the development of some predictive mathematical models [6,11].

The present study focuses on the sedimentation phase, which is considered one of the main loss processes affecting the population dynamics of *Microcystis* [12] and of phytoplankton in general [13,14]. Sedimentation in *Microcystis* spp. occurs when its natural buoyancy, achieved by the presence of intracellular gas vesicles [15] is counteracted by certain ballasts, including the following: (1) the intracellular accumulation of photosynthetic materials (carbohydrates) due to reduced respiration, related to a decrease in water temperature [5,9] and (2) certain particles suspended in water, such as iron-containing colloids [8] and, particularly, clay particles [12], which may aggregate to the mucilage of *Microcystis* colonies and increase their density.

Field studies on lakes and reservoirs have confirmed *Microcystis* spp. sedimentation both during bloom developments and immediately after the disappearance of these pelagic populations [5,6,16]. In general, maximum settling rates are observed during the bloom disappearance and often coincide with the autumnal drop in water temperature [5,9,17]. For instance, Takamura and Yasuno [17] observed a progressive increase in the sinking velocity of *Microcystis* from 0.004 m d^−1^ in June to 0.24 m d^−1^ in October in the shallow Lake Kasumigaura (Japan). A similar maximum velocity (0.25 m d^−1^), equivalent to a settling rate of approximately 0.03 d^−1^ was reported by Verspagen *et al.* [6] in autumn in the shallow areas of Lake Volkerak (The Netherlands). Other studies on *Microcystis* have reported higher maximum settling rates, such as 0.11 d^−1^ in the deep Lake Mendota, USA [16] or even 0.16 d^−1^ in the shallow Lake Crose Mere, UK [18] at certain moments. However, the causes for these increased settling rates could not be clearly determined. 

In addition to water characteristics (temperature and concentration of suspended particles), factors intrinsic to *Microcystis* colonies might also influence their sedimentation dynamics. Analyzing such biological factors is especially interesting if considering that colonies within a single *Microcystis* bloom often differ widely in morphology (e.g., different colony sizes and shapes), physiological status and chemical properties [19,20]. According to several mathematical models, the colony diameter influences vertical migration during daily sinking-ascending cycles of *Microcystis* [21,22] with large colonies putatively showing higher sinking and ascending velocities than smaller ones. However, the influence of colony size on irreversible sedimentation during or after blooms remains poorly understood. The physiology and metabolic activities of colonies might also play a role in sedimentation, as the loss of buoyancy in colonies has been linked to low efficiency in carbohydrate metabolism or to the formation of intracellular polyphosphate bodies [23]. *Microcystis* sedimentation has also been associated with an increase in dead cells within settling *Microcystis* colonies compared with those in the upper epilimnion, suggesting that programmed cell death could precede the sedimentation phase [24]. Concerning the chemical diversity of colonies, the few studies on the dynamics of intracellular MC content in benthic *Microcystis* have indicated similar cell quotas of MCs [5,25] and profiles of MC variants [20,26] in benthic and pelagic populations, however, possible shifts in colonies during sedimentation have not been specifically addressed. 

*Microcystis* spp. are common in Spanish freshwater reservoirs, as found in 16 of the 47 Spanish reservoirs surveyed by De Hoyos *et al.* [27] or in the seven reservoirs of the Madrid area (Central Spain) investigated by Carrasco *et al.* [28]. In the latter study, *Microcystis* dominance occurred mainly in July, September and October, and generally correlated with high toxin concentrations, such as the 70 μg MC L^−1^ reached in the Santillana reservoir [28]. Wörmer *et al.* [26] investigated the settling rates of the MC toxins during *Microcystis*-dominated blooms in the Cogotas, Santillana and Valmayor reservoirs (Central Spain) and found that, on average, 4.5% of the pelagial toxins were settling daily during such blooms. Interestingly, Wörmer *et al.* also observed that the sestonic MC:organic matter ratio decreased in the hypolimnetic sediment traps when compared with the epilimnetic sediment traps, suggesting a decrease in the MC cell quota in the settled cells. 

Based on the same experimental setup used by Wörmer *et al.* [26], the present study focused on the sedimentation dynamics of *Microcystis* populations in three water reservoirs (Cogotas, Santillana and Valmayor) in Central Spain. The following aims were investigated: (1) determine the quantitative importance of the sedimentation process in the loss of pelagic *Microcystis* populations; (2) establish the spatiotemporal patterns of the sedimentation processes, evaluating the influence of environmental factors (temperature and inorganic matter content) and/or colony morphology (morphospecies and colony size) on such patterns; and (3) monitor the shifts in the MC cell quotas of settling *Microcystis* populations.

## 2. Results and Discussion

### 2.1. Microcystis and MCs in Water

The three reservoirs studied are characterized in Table 1. According to the temperature profiles (data not shown), the water column was thermally stratified in Santillana from the beginning of the sampling period until the first half of October, whereas in Valmayor, the water column started to mix at the end of September and was fully mixed on 15 October. In Cogotas, a massive water withdrawal occurred during the summer of 2006 with a reduction in depth to less than 15.3 m, resulting in the mixing of the water column. During stratification, the upper limits of the thermocline were placed at 7 m in Valmayor and 6 m in Santillana. Further details on the thermal structure of the water columns in the three reservoirs can be found in [26].

The three reservoirs developed cyanobacterial blooms during the study period, which occurred during the whole sampling period in Cogotas, from mid-August to mid-November in Santillana and from the beginning of the sampling (30 August) to 29 October in Valmayor.

**Table 1:** Characteristics of the water reservoirs under investigation. ^a^ Due to massive water withdrawal, the depth of the water column was drastically reduced; maximum depth observed was 15.3 m; ^b^ D: drinking; R: recreational; I: irrigation.

Reservoir	Watershed	River	Depth (m)		Water uses ^b^
			Mean	Maximum	
Cogotas	Duero	Adaja	14.9 ^a^	60 ^a^	D, I
Santillana	Tajo	Manzanares	8.7	36	D
Valmayor	Tajo	Aulencia	16.4	51	D, R

**Figure 1:**
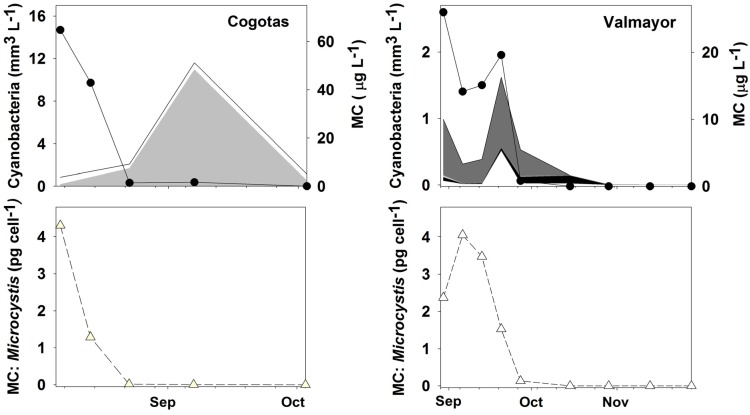
Bloom dynamics and MCs in the Cogotas and Valmayor reservoirs. Cyanobacteria are represented in the top graphs by area plots: *Microcystis aeruginosa* (dark grey); *Microcystis flos-aquae* (light grey); *Microcystis novacekii* (black); others (white). MC concentration (sum of MC-LR, MC-RR and MC-YR) is represented by black circles and a solid line. Estimated MC cell quota (sum of MC-LR, MC-RR and MC-YR) in *Microcystis* is represented in the bottom graphs by white triangles and a dashed line.

**Figure 2:**
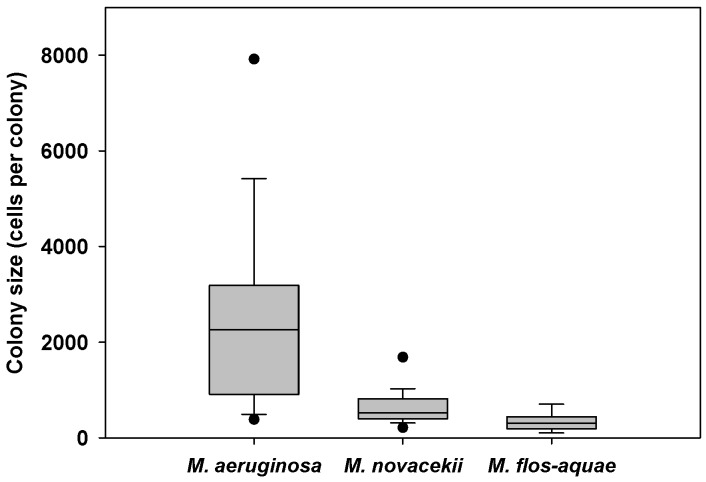
Colony sizes of *Microcystis* morphospecies in subsurface water at the Valmayor reservoir (box-plots). Dots represent the 5th and 95th percentiles.

*Microcystis* spp. dominated Cogotas and Valmayor phytoplankton during most of the studied period (Figure 1), whereas Santillana showed a lower presence of this genus (data not shown). In Cogotas, *M. flos-aquae* dominated the cyanobacterial community from 14 August onwards, accounting for 21%–94% of the cyanobacterial biovolume (developing massively on 7 September with 487,000 cells mL^−1^). In Santillana, *M. flos-aquae* dominated the community prior to the studied period (7500 cells mL^−1^ on 17 July, data not shown) but then showed a moderate presence of 500–3000 cells mL^−1^ during the study. *M. aeruginosa* appeared only at low levels (below 500 cells mL^−1^) in Santillana. In Valmayor, three *Microcystis* morphospecies were identified: *Microcystis aeruginosa*, which dominated from August to 15 October and reached 12,170 cells mL^−1^; *Microcystis novacekii*, which dominated from 15 October to 29 October with 1500 cells mL^−1^ as maximum; and *Microcystis flos-aquae*, which accounted for less than 2.6% of the *Microcystis* biovolume and was below 500 cells mL^−1^ during the study period. The marked morphological diversity of the *Microcystis* community in Valmayor was also reflected in the wide range of colony sizes (Figure 2) with *M. aeruginosa* showing the broadest range of colony sizes.

The MC hepatotoxins (MC-LR, MC-RR, MC-YR) were present in Cogotas and Valmayor but were not detected in Santillana (see [26] for more details). *Microcystis* spp. and MC dynamics were intimately linked in Valmayor (Figure 1). Indeed, the MALDI-TOF/MS analyses that were performed on 100 single *Microcystis* colonies from Valmayor (data not shown) confirmed MC production by *M. aeruginosa* and *M. flos-aquae*. In Cogotas, the high MC concentrations reached on 7 August (64.8 μg MC L^−1^) and 14 August (42.9 μg MC L^−1^) coincided with low *M. flos-aquae* concentrations, whereas the opposite occurred from 23 August onwards. Despite this apparent lack of a relationship, MC production was confirmed by HPLC-PDA in a *M. flos-aquae* strain (UAM 297) isolated from Cogotas on 4 August. MC cell quotas (pg cell^−1^) estimated for *Microcystis* spp. showed sudden variations in both Cogotas and Valmayor (Figure 1) with maxima found during the early stages of *Microcystis* bloom development. For example, 1.2–4.3 pg MC cell^−1^ in Cogotas on 7–14 August and 3.4–4.1 pg MC cell^−1^ in Valmayor on 6–13 September were found followed by a drop to 1.2 pg MC cell^−1^ and a more marked decrease to 0.01 pg MC cell^−1^ during subsequent *Microcystis* peaks in Valmayor (20 September) and Cogotas (9 September), respectively. This finding is in agreement with the frequent observation of toxicity peaks coinciding with the first stages of *Microcystis* bloom development [29] due to yet undetermined causes.

### 2.2. Settling Dynamics of Microcystis

The present study investigated the settling dynamics of *Microcystis* in three water bodies in Central Spain, a geographical area (Mediterranean Europe) in which this genus is considered the main MC producer [28,30]. We therefore add novel data to previous works on *Microcystis* settling in lakes of the USA [16], Japan [17] and Central-Northern Europe [5,6,18].

#### 2.2.1. Spatiotemporal Patterns

*Microcystis* cells were found in the sediment traps of the three reservoirs during the whole study period (Table 2).

**Table 2:** Settling fluxes of *Microcystis* in the three reservoirs studied. ^a^ The ratio between *Microcystis* dry weight (*Mic*) and organic matter (OM) was estimated from the *Microcystis* settling rate (cells m^−2^ d^−1^) and an average of 2.04 × 10^−5^μg dry weight cell^−1^ [31]; ^b^ Data in parentheses indicate the % of total *Microcystis* spp. biovolume.

Reservoir		Date	Total *Microcystis* settled			Dominant species ^b^
			10^9^ cells m^−2^ d^−1^	mm^3^ m^−2^ d^−1^	*Mic*: OM (%) ^a^	
Cogotas	Epilimnetic	14/08/2006	13.4 ± 3.1	300.4 ± 69.2	3.8	*M. flos-aquae* (100%)
		23/08/2006	18.2 ± 0.9	409.3 ± 21.7	4.2	*M. flos-aquae* (100%)
		07/09/2006	36.7 ± 0.7	823.4 ± 16.8	7.6	*M. flos-aquae* (100%)
		03/10/2006	17.3 ± 9.1	389.2 ± 203.7	5.3	*M. flos-aquae* (100%)
Santillana	Epilimnetic	01/08/2007	0.07 ± 0.04	1.5 ± 0.8	0.1	*M. flos-aquae* (100%)
		13/08/2007	0.07 ± 0.008	1.4 ± 0.2	0.1	*M. flos-aquae* (100%)
		29/08/2007	-	-	-	*-*
		11/09/2007	0.001 ± 0.001	0.01 ± 0.008	0.002	*M. flos-aquae* (100%)
		05/10/2007	0.001 ± 0.001	0.03 ± 0.005	0.003	*M. flos-aquae* (100%)
		22/10/2007	0.001 ± 0.001	0.02 ± 0.008	0.002	*M. flos-aquae* (100%)
	Hypolimnetic	01/08/2007	0.1 ± 0.04	2.3 ± 0.8	0.1	*M. flos-aquae* (100%)
		13/08/2007	0.2 ± 0.08	4.7 ± 1.9	0.3	*M. flos-aquae* (87%)
		29/08/2007	-	-	-	-
		11/09/2007	-	-	-	-
		05/10/2007	0.03 ± 0.004	0.6 ± 0.1	0.05	*M. flos-aquae* (100%)
		22/10/2007	0.008 ± 0.001	0.2 ± 0.02	0.005	*M. flos-aquae* (100%)
Valmayor	Epilimnetic	06/09/2007	2.9 ± 0.5	209.0 ± 37.8	2.2	*M. aeruginosa* (98%)
		13/09/2007	0.7 ± 0.06	50.3 ± 4.1	0.5	*M. aeruginosa* (99%)
		27/09/2007	3.4 ± 0.3	190.3 ± 19.6	2.8	*M. aeruginosa* (86%)
		15/10/2007	0.4 ± 0.2	27.0 ± 13.6	0.3	*M. aeruginosa* (84%)
		29/10/2007	1.2 ± 0.2	52.4 ± 6.5	1.2	*M. novacekii* (55%)
		28/11/2007	0.05 ± 0.008	3.0 ± 0.5	0.02	*M. aeruginosa* (81%)
	Hypolimnetic	06/09/2007	1.6 ± 0.6	101.8 ± 34.8	0.9	*M. aeruginosa* (98%)
		13/09/2007	1.5 ± 0.6	91.0 ± 37.5	1.2	*M. aeruginosa* (99%)
		27/09/2007	4.2 ± 0.1	295.4 ± 2.5	1.2	*M. aeruginosa* (98%)
		15/10/2007	2.6 ± 0.4	129.7 ± 0.02	1.0	*M. aeruginosa* (69%)
		29/10/2007	1.1 ± 0.01	50.9 ± 4.5	0.7	*M. aeruginosa* (58%)
		28/11/2007	0.08 ± 0.02	5.2 ± 1.3	0.05	*M. aeruginosa* (96%)

The settling fluxes exceeded 10^6^ cells m^−2^ d^−1^ in the three reservoirs, and were even above 10^9^ cells m^−2^ d^−1^ on certain dates at Valmayor, and in all the epilimnetic traps at Cogotas, where a maximum of 3.7 × 10^10^ cells m^−2^ d^−1^ settled between 23 August and 7 September. In Santillana, much lower fluxes were observed, with a maximum (when expressed as biovolume) of two orders of magnitude below the levels measured at Valmayor or Cogotas. The “*Microcystis* rain” observed in the three reservoirs was estimated to account for up to 0.1%, 2.8% and 7.6% of the organic matter settled in traps at Santillana, Valmayor and Cogotas, respectively. Fallon and Brock [16] reported that a 0.5%–1% of the organic matter in traps came from *Microcystis*-dominated phytoplankton that had settled in Lake Mendota, USA, which is in the range of the 0.1%–2.8% we found for Valmayor, but is clearly below the 3.8%–7.6% estimated for the Cogotas reservoir. However, the results found at Cogotas may be overestimated, because the average dry weight per cell used (derived from [31]) was taken from a *M. aeruginosa* strain, whose cells are often bigger than those of *M. flos-aquae* [32], the only *Microcystis* found in Cogotas traps. Therefore, when *Microcystis* dominates the phytoplankton for certain periods, it is expected to provide for about 1% (and eventually more) of the organic matter settled in lakes and reservoirs, reflecting its importance in the mass fluxes between water and the sediments of such water bodies.

Table 3 shows the settling rates (d^−1^) estimated for the whole *Microcystis* population in the Valmayor reservoir.

**Table 3:** Estimated settling rates (d^−1^) of *Microcystis* spp. at the Valmayor reservoir. The results are expressed as the mean ± standard deviation.

Layer	Period					
	1–6 September	6–13 September	13–27 September	27 September–15 October	15–29 October	29 October–28 November
Epilimnion	0.05 ± 0.009	0.03 ± 0.002	0.04 ± 0.004	0.01 ± 0.006	0.07 ± 0.009	0.04 ± 0.006
Entire water column	0.02 ± 0.009	0.04 ± 0.02	0.06 ± 0.001	0.05 ± 0.01	0.05 ± 0.004	0.05 ± 0.003

The settling rates fluctuated without a clear temporal pattern. In general, the average settling rates after column mixing (15 October) were higher than the pre-mixing rates for the epilimnion (0.031 d^−1^ before 15 October and 0.055 d^−1^ after 15 October) and for the hypolimnion (0.043 d^−1^ before 15 October and 0.048 d^−1^ after October 15), although such differences were not statistically significant (*t*-test; *p* > 0.05). The epilimnetic rates were markedly increased from 15–29 October, reaching 0.07 d^−1^. Nevertheless, this rise was not reflected in the hypolimnion either during the same period or in the subsequent period (29 October–28 November). As a whole, the epilimnetic rates averaged 0.039 ± 0.021 d^−1^, whereas the hypolimnetic rates were more homogeneous, averaging 0.044 ± 0.013 d^−1^. Therefore, approximately 4%−4.4% of the *Microcystis* colonies were settling at any given moment in the Valmayor reservoir between September–November 2007. This percentage was equivalent to an average settling velocity of 0.7 m d^−1^ for the 16-m water column, with a maximum of 0.96 m d^−1^ at the end of September. In other words, *Microcystis* colonies could reach 16 m in approximately 22 days (at 0.7 m d^−1^) or even in 17 days (at 0.96 m d^−1^) during certain periods. These data are in good agreement with findings by Wörmer *et al.* [26] in Valmayor in September 2007, in which an average of 4.5% of settled sestonic toxins (MCs) was reported at any given time-point, and a time lag of two-three weeks was estimated for those molecules to descend the water column within (most likely) intact *Microcystis* colonies. Maximum *Microcystis* settling velocities in Valmayor (0.7–0.9 m d^−1^) were markedly higher than those reported in most previous studies, such as the maximum of 0.24 m d^−1^ and 0.25 m d^−1^ estimated in lakes Kasumigaura, Japan [17] and Volkerak, the Netherlands [6], but (if expressed as d^−1^) were in the range of the maximum 0.11 d^−1^ observed in Lake Mendota, USA [16] or the 0.16 d^−1^ reported in Rostherne Mere, UK [18] at particular moments. These data indicate that the settling velocity of *Microcystis* varies among water bodies, ranging from <0.1 m d^−1^ to approximately 1 m d^−1^, and may explain the <1% to above 5% (or even 10%) of the losses of pelagic *Microcystis* per day. Previous work has suggested that a drop in water temperature [5,9] and/or the adsorption of inorganic particles to *Microcystis* colonies [8,12] are triggers for sedimentation. However, we did not find a global correlation (Spearman correlation test; *p* > 0.05) between water temperature (average water temperature during each settling period) and settling rates of *Microcystis* spp. (d^−1^) or between settling rates of inorganic matter (g m^−2^ d^−1^) and settling rates of *Microcystis* spp. for the epilimnion or for the whole water column. Another commonly observed trend is that *Microcystis* settling starts during summer blooms and increases markedly after bloom disappearance in autumn [5,9,17]. In Valmayor, the settling rates during the progressive disappearance of the *Microcystis* bloom (15 October–28 November) were only 1.7-fold (epilimnion) and 1.1-fold (hypolimnion) higher than those measured during the bloom (30 August–15 October). This finding contrast with the 10-fold increase in October (post-bloom) settling rates compared with those of September (during the bloom) observed in shallow Lake Kasumigaura [17]. The lack of a sudden increase in settling after the bloom in Valmayor might have several explanations. First, it is possible that almost all the settling rates we reported in Valmayor were already included in the “autumnal sedimentation” phase assumed elsewhere, as the water temperature range at the Valmayor epilimnion (11.2–19.4 °C) was within the range of temperatures triggering sedimentation reported by previous studies [5], and references therein. This is also in agreement with our observations of homogeneously high *Microcystis* settling rates that showed no global correlation with temperatures in Valmayor. Secondly, hypothetical differences between average settling rates may be lessened by the low number of post-bloom rates (*n* = 2) as well as by the uncertainty of the sources inherent in the trap sampling study. For instance, some hypolimnetic settling rates during the bloom might be overestimated by re-suspension events that were not measured, such as those that most likely occurred on 27 September [26]. Conversely, the last post-bloom rate of 0.05 d^−1^ (29 October–28 November) might be an underestimate because of possible losses of biomass in traps due to increased grazing and/or viral or bacterial decomposition during the long 29-day settling period.

#### 2.2.2. *Microcystis* Morphospecies

The *Microcystis* morphospecies detected in traps coincided with those observed in water from the 3 reservoirs (Table 2). *M. flos-aquae* was the most abundant morphospecies found in traps at Cogotas and Santillana with *M. aeruginosa* appearing only in the hypolimnetic traps at Santillana on 13 August. *M. aeruginosa* was the most abundant morphospecies found in Valmayor traps. There, the shift in dominance from *M. aeruginosa* to *M. novacekii* that occurred in subsurface water from 27 September onwards was clearly reflected in the epilimnetic traps (*M. novacekii* represented 55% of *Microcystis* biovolume on 29 October) and less evidently in the hypolimnetic traps.

Table 4 shows the settling rates for each morphospecies, estimated following the same approach as that used for the whole *Microcystis* community. *M. aeruginosa* showed the highest average settling rate for both the epilimnion (0.033 d^−1^) and for the first 16 m (0.053 d^−1^), although the differences with *M. flos-aquae* and *M. novacekii* were only significant for the whole water column (one-way ANOVA followed by post-hoc Holm-Sidak test; *p* < 0.05) but not for the epilimnion (*p* > 0.05). Estimated settling velocities of *M. aeruginosa* reached 1.1 m d^−1^ at certain moments, indicating that some colonies might reach 16 m in approximately 2 weeks.

**Table 4:** Estimated settling rates of *Microcystis* morphospecies at the Valmayor reservoir during the entire study period. SD: standard deviation.

Layer	Morphospecies	Estimated settling rate (d^−1^)		Estimated settling velocity (m d^−1^)	
		Average ± SD	Range	Average ± SD	Range
Epilimnion	*M. aeruginosa*	0.033 ± 0.014	0.019–0.078	0.3 ± 0.1	0.2–0.8
	*M. flos-aquae*	0.010 ± 0.005	0.004–0.017	0.1 ± 0.05	0.0–0.2
	*M. novacekii*	0.030 ± 0.028	0.005–0.080	0.3 ± 0.3	0.1–0.8
Entire column	*M. aeruginosa*	0.053 ± 0.021	0.024–0.071	0.8 ± 0.3	0.4–1.1
	*M. flos-aquae*	0.024 ± 0.015	0.010–0.042	0.4 ± 0.2	0.1–0.7
	*M. novacekii*	0.012 ± 0.011	0.006–0.029	0.2 ± 0.2	0.1–0.5

**Figure 3:**
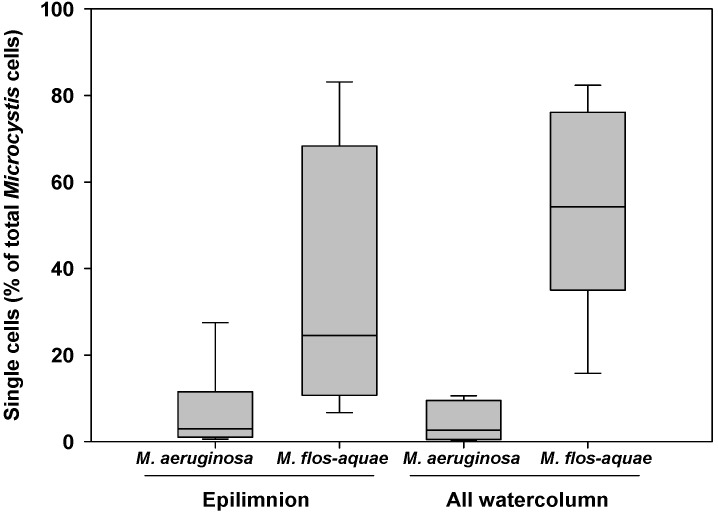
Box-plots representing the number of single *Microcystis* cells among the total number of *Microcystis* cells settled in two depths of the Valmayor reservoir.

Figure 3 shows the percentage of free cells (cells not grouped within the mucilaginous envelope) of *M. aeruginosa* and *M. flos-aquae* in Valmayor. Although there was considerable inter-sample dispersion, settled *M. flos-aquae* showed a significantly higher percentage of free cells than *M. aeruginosa* for both the epilimnion and for the whole water column (Mann-Whitney rank sum test; *p* < 0.05). The differences for the entire water column (16 m) were more marked with *M. flos-aquae*, which showed a median of 56% and a range of 16%–82% of free cells in comparison with a median of 5% and a range of 0.1% to 11% of free cells estimated in *M. aeruginosa* for the same layer. 

Most previous field studies have focused either on *M. aeruginosa* or on the *Microcystis* community as a whole without distinguishing between *Microcystis* morphospecies. We found a higher hypolimnetic settling of *M. aeruginosa* than of *M. novacekii* and *M. flos-aquae* in the Valmayor reservoir. A trend of an increased release of cells was also observed in settled *M. flos-aquae* compared with settled *M. aeruginosa* upon microscopic observations of Cogotas traps (Figure S1 in supplementary material). We hypothesize that the reduced settling velocities of *M. flos-aquae* at Valmayor may be related to a progressive disintegration of colonies into single cells, generally more susceptible to grazing losses than integer colonies. However, this phenomenon may vary among grazers as amoebas feed more easily on colonies than on single *Microcystis* cells [33].

Regarding *M. aeruginosa* in Valmayor, many of the large colonies (100–1000 μm long) observed in 16-m water in September were characteristically emptied; although their mucilaginous envelope was maintained, a much lower density of cells was observed here than in the subsurface water (Figure S1 in supplementary material). Similarly, Sigee *et al.* [24] observed a high proportion of disintegrated *M. flos-aquae* colonies within sediment traps and reported over 30% of dead cells in many epilimnetic colonies, suggesting that programmed cell death precedes the major phase of sedimentation. Whether the trends we observed in *M. aeruginosa* and *M. flos-aquae* are species-specific or strain-specific and their possible causes (e.g., programmed cell death) remain to be determined.

#### 2.2.3. Colony Sizes

The colony diameter is considered a critical factor in determining the vertical movements of *Microcystis* colonies in daily cycles during blooms [21,22]; however, information on the influence of diameter on irreversible sedimentation is scarce.

In addition to the differences between species, we assessed whether the three colony classes established for Valmayor (<1000 cells; 1000–5000 cells; >5000 cells; see Figure 2) had different settling rates (Table 5).

**Table 5:** Estimated settling rates of *Microcystis* colonies of different sizes at the Valmayor reservoir during the entire study period. SD: standard deviation.

Layer	Size class (cells per colony)	Estimated settling rate (d^−1^)		Estimated settling velocity (m d^−1^)	
		Average ± SD	Range	Average ± SD	Range
Epilimnion	*<*1000	0.034 ± 0.034	0.010–0.093	0.3 ± 0.3	0.1–0.9
	1000–5000	0.037 ± 0.030	0.005–0.080	0.4 ± 0.3	0.1–0.8
	>5000	0.043 ± 0.032	0.010–0.078	0.4 ± 0.3	0.1–0.8
Entire watercolumn	*<*1000	0.043 ± 0.020	0.006–0.064	0.7 ± 0.3	0.1–1.0
	1000–5000	0.036 ± 0.022	0.018–0.067	0.6 ± 0.4	0.3–1.1
	>5000	0.039 ± 0.024	0.021–0.074	0.6 ± 0.4	0.4–1.2

No significant differences were found in the average settling rates of the three size classes for either the epilimnion or the whole water column (one-way ANOVA; *p* > 0.05), likely reflecting the large standard deviations observed. The ranges were especially wide in the epilimnion and were in accord with the marked differences observed between dates. In the small (<1000 cells) and medium-sized (1000–5000 cells) colonies, such fluctuations appeared to be related to the temporal pattern, because the rates prior to column mixing (on 15 October) were generally lower (0.010–0.024 d^−1^ for the small colonies; 0.005–0.046 d^−1^ for the medium-sized colonies) than those obtained after 15 October (0.028–0.093 d^−1^ for the small colonies; 0.058–0.080 d^−1^ for the medium-sized colonies). Large colonies (>5000 cells) disappeared from the subsurface of the water before 15 October, thus the trend could not be analyzed. Still, during the bloom period, the epilimnetic settling rates of large colonies between 30 August and 13 September (0.061–0.078 d^−1^) were markedly higher than those observed between 13 September and 15 October (0.010–0.023 d^−1^). For the hypolimnion, no clear temporal trend was observed. The velocities estimated for the colony sizes showed an overall range of 0.1–1.2 m d^−1^, which resembled the 0.1–1.1 m d^−1^ range estimated for the different *Microcystis* species (Table 4). Both the average velocities and their ranges were similar in the three size classes for the whole water column with overall settling rates of 0.036–0.043 d^−1^. However, some size-dependent trends were observed. Large colonies (>5000 cells per colony) could exhibit extremely high settling rates (0.074 d^−1^ during the 6–13 September) or low rates (0.021 d^−1^ during the 13–27 September) during blooms, and such fluctuations may be related to sudden changes in colony buoyancy. For instance, the apparently highly buoyant subsurface population of large *M. aeruginosa* colonies observed on 20 September, which was drastically reduced (80% less) one week later (27 September), was followed by the lowest settling rates during the subsequent periods of 13–27 September (0.021 d^−1^) and 27 September–15 October (0.037 d^−1^). We hypothesize that most of this over-buoyant population disappeared by the formation (and subsequent degradation) of surface scums that were indeed observed in Valmayor during the month of September. Another trend suggested by our data was that small (<1000 cells per colony) and medium-sized (1000–5000 cells per colony) colonies were apparently more affected by water temperature decreases or column mixing as reflected by higher average settling rates from 15 October on than before this date. Our results on settling can be linked to those of the mathematical model on the daily movements of *Microcystis* by Visser *et al.* [21]. These authors predicted that large colonies may show both the highest flotation and sinking velocities and that the small colonies have a higher increase in net cell density, which may make them more prone to irreversible sedimentation due to, for instance, a drop in water temperature.

### 2.3. Shifts in MC Cell Quota during Settling

Table 6 shows the estimated MC cell quotas in *Microcystis* cells in water and sediment traps in the three reservoirs. It must be noted that quotas were obtained by dividing the whole sestonic MC concentration by the number of *Microcystis* cells without considering any of the other putative MC-producing species or non-MC producing *Microcystis* strains.

**Table 6:** Comparison of the estimated MC cell quotas in *Microcystis* from water and sediment traps in the three reservoirs studied. MC content is expressed as the average and range (in parentheses) of all the sampling dates within the period (*n* = 4 in Cogotas, *n* = 2 in Santillana, *n* = 5 in Valmayor). Nd, not detected.

Reservoir	Period	Sample	MC content (pg MC *Microcystis* cell^−1^)
Cogotas	7/08/2006–7/09/2006	Subsurface water	1.4 (0.01–4.3)
		Epilimnetic trap	0.1 (0.01–0.2)
Santillana	1/08/2007–13/08/2007	Subsurface water	Nd
		Epilimnetic trap	0.15 (0.14–0.15)
		Hypolimnetic trap	0.10 (0.09–0.10)
Valmayor	30/08/2007–27/09/2007	Subsurface water	2.3 (0.1–4.0)
		Epilimnetic trap	0.6 (0.8–1.2)
		Hypolimnetic trap	0.4 (0.3–0.6)

MC cell quota in traps was in general lower than that of the subsurface water in both the Cogotas and Valmayor reservoirs, whereas in Santillana, MCs in water remained below the detection limit but the toxins were found in low amounts in traps (see [26] for more details on MC dynamics). The average cell quotas in epilimnetic traps were 14-fold lower than those in the subsurface water in Cogotas, whereas the difference was smaller (4-fold) in Valmayor. In addition, the maximum cell quotas were lower in epilimnetic traps than in water in both Cogotas (20-fold lower) and Valmayor (3-fold lower). Regarding the hypolimnetic traps, both the average and the maximum cell quotas were slightly below those of the epilimnetic traps, averaging approximately 1.5-fold lower in hypolimnetic traps compared with epilimnetic traps in both the Santillana and Valmayor reservoirs.

The 4–14 fold reduction in the average cell quota of MCs in settling *Microcystis* of Cogotas and Valmayor might have different causes. One possibility is the differential settling of *Microcystis* chemotypes, with an increased settling of non-MC producing and/or low MC-containing colonies. In Valmayor, single-colony MALDI-TOF/MS analyses performed on a water sample taken on 6 September, 2007 suggested the existence of at least five different chemotypes of *M. aeruginosa* (including both MC-containing and non-containing colonies) [34], and these chemotypes could be hypothetically settling at different rates. In the Brno reservoir, Welker *et al.* [20] observed that although the benthic *Microcystis* population in November contained over 90% of the pelagic chemotypes of the previous summer, the relative proportion of certain chemotypes varied greatly between benthic and pelagic populations, suggesting a differential survival of chemotypes during settling and/or benthic phases. A second possibility is that similar settling rates take place in different chemotypes but a decrease in the MC cell quota occurs due to processes that may happen during descent, such as internal consumption of MC and/or new synthesis (after cell division) of non-MC and/or low MC-containing cells in descending colonies. Some authors have proposed the self-consumption of MCs by *Microcystis* cells [35] or the use of MCs as internal nitrogen sources [36]. Other groups have suggested that MCs are degraded by bacteria from *Microcystis* mucilage [37], a process that could be enhanced during the *Microcystis* descent along the Valmayor water column. A third and very interesting option, based on recent findings by Zilliges *et al.* [38], is that an increase in protein-bound MCs in senescent settling *Microcystis* cells occurs. The latter study reported that MCs are covalently bound to certain proteins, especially under stress conditions, and such binding could result in an apparent decrease in MC quotas, as covalently-bound MCs might not be measured after the methanol extraction used in our protocol. The decrease in MC cell quotas during settling reported here contrasts with recent observations during *Microcystis* recruitment, including the selection of *mcy*^+^ genotypes [39] and the increase in MC content [40] found in recruited cells compared with the initial benthic stock. Whether MC dynamics during settling are the inverse of those during recruitment and the true causes of the decrease during settling remain to be clarified by further studies. 

## 3. Experimental Section

### 3.1. Sampling Setup

The study was performed in three reservoirs located in Central Spain: the Cogotas reservoir in 2006 and the Santillana and Valmayor reservoirs in 2007. Their main characteristics are shown in Table 1. The reservoirs were monitored from June to November, but intensive sampling started only when bloom development was evident and lasted until the *Microcystis* colonies were absent from the epilimnetic water. Thus, sampling periods were 7 August–3 October in Cogotas, 25 July–22 October in Santillana and 30 August–28 November in Valmayor. Sampling was performed between 11 a.m. and noon at weekly or fortnightly intervals.

On the first sampling date, one sampling point was located in the deep area of each of the reservoirs and was marked with a buoy. On the same date, sediment traps were placed in the same sampling points. These traps were designed in the laboratory and constructed by SEGAINVEX (Universidad Autónoma de Madrid). Each trap set consisted of three PVC cylinders (4.4 cm internal diameter) that were wrapped with black tape to avoid growth of photosynthetic organisms. The traps were attached to two buoys, which were fixed by two anchors. A central weight allowed further stabilization. The traps were thus freely suspended in the water column. One trap set was placed in the upper metalimnion in the three reservoirs and another was placed in the hypolimnion (namely, 1 m above the sediment surface) of the Santillana and Valmayor reservoirs. In Cogotas, the massive water withdrawal that took place in 2006 resulted in water column mixing and a reduced depth during the sampling period: thus only the epilimnion was studied.

### 3.2. Water Column Sampling

Vertical profiles of temperature, chlorophyll *a* (Chl *a*) and dissolved oxygen were obtained with an YSI 6920 multiparameter probe on each sampling date. Water samples were then taken at a 0.5-m depth, and at the depths where traps were deployed, with the aid of a 5-L water sampler (Uwitec, Mondsee, Austria). Water was transported to the lab at 4 °C and processed within two hours. Once the water samples reached room temperature, Chl *a* concentration (μg L^−1^) and algal group composition were determined using a benchtop fluorometer (Moldaenke BBE Algae Analyser, Schwentinental, Germany). A 100-mL aliquot was fixed with acid lugol and kept in the dark at 4 °C until subsequent microscopic analysis. The remaining water was low-vacuum filtered through GF/F glass fiber filters (Whatman, Kent, UK) that were kept at −20 °C for MC analysis.

### 3.3. Sediment Trap Sampling

Trap sampling took place simultaneously with the water column sampling at the same weekly or fortnightly intervals. On each sampling date, settled material in the traps was recovered by discarding the water supernatant in each tube and collecting the 100 mL (including sediment) remaining in the trap. No *Microcystis* colonies were observed in the removed supernatants. After homogenization of the settled matter, aliquots were taken for quantification of organic matter and inorganic matter content, identification and quantification of settled *Microcystis* and quantification of particle-associated MCs. All measurements were performed individually for each trap, and the results shown are average values of three replicates. Organic matter (OM) and inorganic matter (IM) were quantified by obtaining the dry weight of the aliquots after desiccation (100 °C, 24 h) and combustion (500 °C, 4 h) in porcelain crucibles. OM was calculated as the difference between desiccated and combusted weights. A second aliquot (10–20 mL) was diluted 10-fold with GF/F filtered water from the trap depth, fixed with 4% formaldehyde (*v*/*v*) and stored at 4 °C in the dark for the identification and quantification of *Microcystis* by epifluorescence microscopy. A third aliquot (10–20 mL) was low-vacuum filtered through GF/F filters and stored at −20 °C for MC analysis. The average sedimentation rates for each period were calculated by relating settled matter to the time elapsed between samples and the surface of the trap openings. The gaps in the data from Santillana are due to the loss of sediment traps on 28 August and 11 September.

### 3.4. Identification and Quantification of Microcystis in Water and Sediment Traps

Identification and quantification of planktonic cyanobacteria in water was performed in lugol-fixed samples that were sedimented following Utermöhl’s procedure [41]. Species identification followed [32,42,43,44]. For quantification, individual cells were counted under an inverted microscope Leica DM IL (Leica Microsystems, Wetzlar, Germany) at 400× magnification until statistically significant numbers of counting units were reached. The number of cells per colony was calculated by counting at least 200 cells per colony and extrapolating the counts to the whole colony surface. Average cell biovolumes were estimated by assimilating cells to regular geometric bodies and measuring relevant dimensions of at least 100 cells of each morphospecies.

Determination of *Microcystis* in sediment traps was performed by filtering 1 mL of the formaldehyde-fixed, 10-fold diluted settled matter of each trap (see Section 3.3) through a 0.2-μm pore and 25-mm diameter Anodisc membrane filter (Whatman, Kent, UK) under low vacuum to avoid colony disruption. The filter was then mounted on a microscopy slide with a drop of anti-fading Aqua-Poly/Mount medium for coverslips (Polysciences, Inc., Warrington, DC, USA) and analyzed with an Olympus BH-2 epifluorescence microscope equipped with a Leica DC 300F digital camera. The epifluorescence system (BH2-RFCA, Olympus, Tokyo, Japan) consisted of a UV Hg lamp OSRAM Short Arc HBO (OSRAM GmbH, Munich, Germany), an excitation filter BP545, a dicroic mirror DM570 and an emission filter O590; the result was green light excitation and visualization of red autofluorescence emitted by cyanobacterial pigments, mainly phycocyanins and phycoerythrins. The whole filter surface was microscopically checked for the presence of *Microcystis* cells. Micrographs were taken and the cell diameter was measured using the image analysis software Leica Qwin (Leica Microsystems, Wetzlar, Germany). In the Santillana and Valmayor reservoirs, only integer *Microcystis* colonies were included in the analyses. In the Cogotas reservoir, because most of the *M. flos-aquae* population settled as single cells, both colonies and single cells were included in the analyses. For quantification, cells in the whole filter surface were counted at 500× magnification. Average cell biovolumes were calculated as described above. Microscopic observations in Valmayor suggested differences in the patterns of colony disaggregation of *Microcystis* morphospecies. Therefore, we analyzed the percentage of free cells (cells not grouped in colonies within a mucilaginous envelope) of *M. aeruginosa* and *M. flos-aquae* in 12 randomly selected sediment-trap samples of Valmayor (1 per settling period per layer). Single cells were sorted in the different species accorded to cell diameter ranges in water samples. The results on *M. novacekii* were not included in the analyses since the number of free cells in the samples (counting units) was statistically insufficient when the microscopic counts where considered to be Poisson-distributed.

### 3.5. Estimation of Settling Rates in the Valmayor Reservoir

The higher sampling resolution achieved at the Valmayor reservoir allowed us to model the depth-time distribution of the whole *Microcystis* community in the first 16 m (Figure S2) over the whole sampling period. Actual *Microcystis* biovolumes (mm^3^ m^−3^) measured in water and sediment samples (sum of the three *Microcystis* morphospecies present) were smoothed by applying the “Negative Exponential” method (SigmaPlot 11.0 software, Systat, Chicago, IL, USA). The modeled biovolume of *Microcystis* spp. (mm^3^ m^−3^) present in the 16 m-water column at any given moment was area-transformed (mm^3^ m^−2^) to calculate the *Microcystis* spp. biovolume overlying the deep epilimnion and the hypolimnion. *Microcystis* settling fluxes (mm^3^ m^−2^ d^−1^) were then related to the overlying biovolume (mm^3^ m^−2^) to estimate the average *Microcystis* settling rates (d^−1^) for the time period elapsed between the two trap samplings.

### 3.6. MC Analysis

Sestonic MCs (MC-LR, RR and YR) in water and sediment trap samples were extracted into 90% (*v*/*v*) methanol and analyzed by high performance liquid-chromatography (HPLC) with photodiode array (PDA), following the procedure described in [26]. The detection limit was 0.02 μg MC per litre of reservoir water.

## 4. Conclusions

The present study reports the striking sedimentation of 10^6^–10^9^ *Microcystis* cells m^−2^ d^−1^ occurring during and after blooms in three water reservoirs from Central Spain (Cogotas, Santillana and Valmayor). The higher sampling resolution in Valmayor allowed for some interesting conclusions: (1) the *Microcystis* settling rates and velocities obtained (0.7 m d^−^^1^) were above most of the velocities reported elsewhere but were in good agreement with MC settling in the same water body [26]; (2) settling may be morphospecies-specific with *M. aeruginosa* showing a higher settling rate than *M. novacekii* and *M. flos-aquae*, and *M. flos-aquae* colonies suffering an apparent colony disintegration during descent; and (3) size-specific trends were observed, including the extremely high and low settling rates achieved by large colonies during the bloom. The 4–14 fold decrease in the average MC cell quota measured in traps from Valmayor and Cogotas presents challenging questions related to the differential settling of toxic and nontoxic chemotypes or hypothetical MC consumption/binding to proteins during descent. Overall, our results indicate that the well-known morphological and chemical diversity of *Microcystis* communities is reflected in their settling dynamics. Therefore, polyphasic work combining microscopy, genetic tools (e.g., 16S–23S ITS and/or *mcy* genes) and mass spectrometry (e.g., chemotype delimitation by single-colony MALDI-TOF/MS analyses) together with mathematical modeling is essential to trace selection processes that occur during colony sedimentation and assess their influence on the annual dynamics of *Microcystis* and its toxins.

## Supplementary Files

Supplementary File 1 Supplementary Information (PDF, 677 KB)
