# Supplementary material for: Sedimentation Patterns of Toxin-Producing Microcystis Morphospecies in Freshwater Reservoirs
Source: Toxins (Basel). 2013 May 3;5(5):939–57. doi: 10.3390/toxins5050939 (PMC3709271; doi:10.3390/toxins5050939)
Supplement: Supplementary File 1 — Supplementary Information (PDF, 677 KB) [file toxins-05-00939-s001.pdf]

Supplementary Information

Figure S1. Micrographs of *Microcystis* spp. in water and sediment traps from Valmayor, Cogotas and Santillana reservoirs. (**a–c**) *Microcystis aeruginosa*: (**a**) from subsurface water in Valmayor reservoir; (**b**) from a sediment trap in Valmayor reservoir (epifluorescence micrograph); (**c**) from 16-m depth water in Valmayor reservoir; (**d**) *Microcystis novacekii* from subsurface water in Valmayor reservoir; (**e–h**) *Microcystis flos-aquae*: (**e**) from subsurface water in Valmayor reservoir; (**f**) from a sediment trap in Valmayor reservoir; (**g**) from a sediment trap in Cogotas reservoir; (**h**) from a sediment trap in Santillana reservoir. Scale bar indicates 100 µm in (**a**), (**b**), (**d**) and (**g**) and 20 µm in (**c**), (**e**), (**f**) and (**h**).

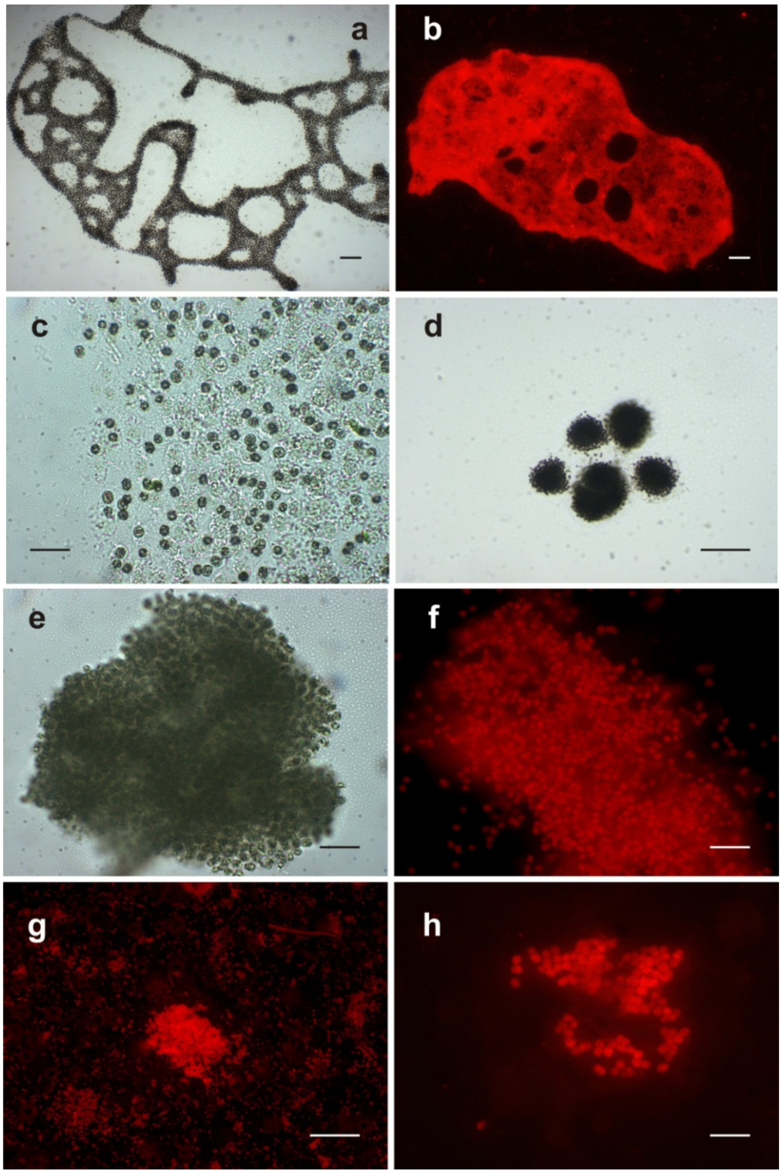

Figure S2. Estimated depth-time distribution of *Microcystis* (mm^3 m^{-3}) in the first 16 m of Valmayor reservoir. White circles indicate actual counts. Data were smoothed by applying the "Negative Exponential" method (SigmaPlot 11.0 software).

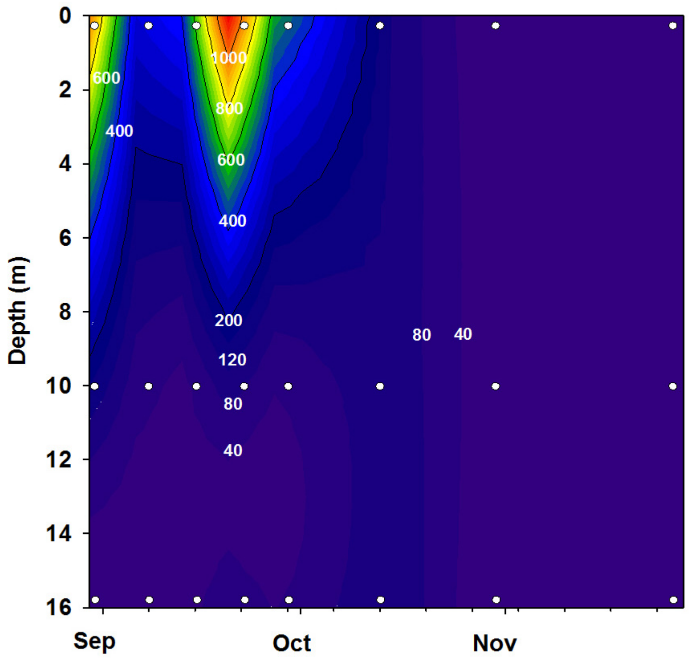

© 2013 by the authors; licensee MDPI, Basel, Switzerland. This article is an open access article distributed under the terms and conditions of the Creative Commons Attribution license (http://creativecommons.org/licenses/by/3.0/).
